# Effective recovery of Ti as anatase nanoparticles from waste red mud via a coupled leaching and boiling route

**DOI:** 10.3389/fchem.2023.1201390

**Published:** 2023-05-18

**Authors:** Zhan Qu, Jiancong Liu, Ting Su, Suiyi Zhu, Junzhen Liu, Yusen Chen

**Affiliations:** ^1^ State Key Laboratory of Pollution Control and Resource Reuse, School of the Environment, Nanjing University, Nanjing, Jiangsu, China; ^2^ Lversheng Environmental Technology Co., Ltd., Chongqing, China; ^3^ Science and Technology Innovation Centre for Municipal Wastewater Treatment and Water Quality Protection, Northeast Normal University, Changchun, China; ^4^ College of Resources and Environment, Zhongkai University of Agriculture and Engineering, Guangzhou, Guangdong, China

**Keywords:** red mud, recycling, anatase, sucrose, economic analysis

## Abstract

Red mud (RM) a solid waste generated by the bauxite smelting industry, is a rich source of metal resources, especially Ti, and its recycling can bring significant environmental and economic benefits. In this study, precious metal Ti was efficiently recovered from red mud using a coupled acid leaching and boiling route for the effective separation of low-value metals. The red mud which contained mainly 10.69% Si, 12.1% Al, 15.2% Ca, 10.99% Fe, and 4.37% Ti, was recovered in five steps. First, a nitric acid solution was used to leach the metals in multiple stages, resulting in an acidic leach solution with high concentrations of Fe, Al, Ti, and Ca ions 2.7 g/L, 4.7 g/L, 5.43 g/L, and 1.8 g/L, respectively. Then, a small amount of sucrose was added as a catalyst to recover Ti from the leach solution under hydrothermal conditions, resulting in the targeted recovery of 98.6% of Ti in the form of high-purity anatase while Fe, Al, and Ca remained in the solution. Next, the Fe in solution was separated as hematite products at a temperature of 110°C and a reaction time of 4 h. Similarly, the Al in the solution was separated and precipitated as boehmite by heating it at 260°C for a reaction time of 20 h. Finally, the remaining Ca in solution was recovered by simple pH regulation. Economic accounting assessment showed that the method yields $101.06 for 1 t of red mud treated, excluding labor costs. This study provides a novel approach to recover precious metals from metal wastes through the whole process resource recovery of solid waste red mud.

## Highlights

1. Facile recovery of 98.6% Ti as anatase from red mud;
2. Ti separation prior to impure Fe, Al, and Ca under atmosphere condition;
3. Separation of Fe and Al as hematite and boehmite prior to Ca;
4. Four purified products of anatase, hematite, boehmite, and gypsum.

## 1 Introduction

As a rare metal with excellent dispersing properties, titanium possesses corrosion resistance and the highest strength-to-weight ratio of any metal. Titanium and its alloys are widely used in the aviation industry, earning them the nickname “space metals,” and are considered strategic metals by many developed countries (Agrawal and Dhawan, 2021; Li et al., 2022). Although titanium is one of the relatively abundant elements in the earth’s crust, the titanium industry, including the production of TiO_2_, currently requires a large amount of titanium resources each year. However, titanium mining and production require a significant amount of energy, raw materials, and chemical reagents. Additionally, due to the complexity of the process, significant equipment and technical support are required, and environmental protection and safety issues need to be considered, all of which increase costs. Therefore, the recovery of titanium resources from waste is a necessary area of research (Fattahpour et al., 2019; Panda et al., 2021). Ti recycling can reduce the need for natural resource extraction, while also reducing energy consumption and emissions and achieving sustainable development of the metals industry, which has significant environmental and economic significance (Qu et al., 2019).

RM is a hazardous industrial solid waste that is generated during alumina production (Wang et al., 2020). However, RM contains valuable metals, such as iron, aluminum, titanium, and rare earth metals, of which the content of Titanium dioxide is relatively high, typically ranging between 4% and 12%. In recent years, China has become the world’s largest producer of alumina, resulting in the annual emission of approximately 100 million tons of RM that requires proper disposal and reuse (Liu et al., 2021; Guo et al., 2022). The current direct disposal methods of RM, which include discharging it into the ocean, wet storage, and dry storage, can cause severe pollution to water, soil, and the atmosphere. Reusing of heavy metal in waste is a hotspot in environmental research, wang et al. reported a novel bionic catalyst obtained from iron-containing biomass, its performance significantly higher than other studies (Bhattacharya et al., 2019; Wang et al., 2023a; Wang et al., 2023b). Therefore, it is crucial to recover these metals using the principles of harmlessness, reduction, and resource recovery (Bhattacharya et al., 2020).

Currently, there are two primary areas of focus in the resource utilization of RM. The first direction involves the bulk utilization of waste, with the goal of reducing the RM stock of enterprises. Related research and development efforts in this area include the reuse of RM for making geopolymer, flocculant and environmental remediation material (Cho et al., 2019; Chao et al., 2022). The second direction focuses on the rich metal elements in RM, particularly trace elements such as Ti, Sr, and Zr. Researchers carry out a series of studies on element leaching, enrichment, and recovery to obtain high-purity by-products and precious metal products (Fei et al., 2021; Chen et al., 2022). In this process, clean separation of Fe/Al/Ca/Ti from the acid solution is the key to achieving efficient utilization of RM resources used high-temperature reductive dissolution separation for Fe/Al enrichment in RM, followed by Al_2_O_3_ enrichment by alkaline leaching of the residue, and finally, Ti recovery using hydrochloric acid leaching. Al and Ti were recovered in the form of sodium aluminum solution and perovskite at a recovery of 85.85% and 95.53% (Li et al., 2020). Yu et al. prioritized the reduction of Fe^3+^ to Fe^2+^ using iron powder based on the leaching of RM with sulfuric acid. Subsequently, the pH was adjusted to less than 5 by adding ammonia to precipitate Al, and then the pH was controlled to be greater than 6 to recover Fe, with insoluble Ti/Si remaining in the solid residue (Yu et al., 2020). Through stepwise separation, high-purity Fe/Al by-products and Ti-containing products can be obtained. Above 90% Fe and Al were extracted from RM with the Fe and Al purity of about 95% and 45%, respectively. The inefficiency of metal recovery, high temperature treatment, a long reaction time, high energy consumption and the lack of product value have been the main reasons why these methods have not been promoted. In contrast, more studies have little or no mention of other elements, and this literature tends to focus on single elements (Kashefi et al., 2019; Russkikh et al., 2020; Yu et al., 2022; Zhang et al., 2022). Accordingly, although good recoveries were obtained for a specific element, the recoveries of other components were little coverage (Narayanan and Palantavida, 2020).

Given the low utilization of RM resources and the urgent need for iron, aluminum, and titanium resources, comprehensive utilization of RM is crucial in the present context (Yu et al., 2020). Thus, this study aims to explore a progressive separation and recovery method for Ti, Fe, Al, and Ca from real RM, thereby converting it from a waste material to a high-value product. Firstly, a multi-stage leaching process is conducted using an acidic solution to extract the metals from the RM. Next, the Ti in the acidic leachate is recovered through a hydrothermal method, which converts it into anatase. Finally, Fe, Al, and Ca are recovered through a hydrothermal method with different parameters and stepwise separation. Overall, this study proposes an innovative and effective method for the disposal and resource utilization of RM, offering a new approach to recover resources from polymetallic solid waste.

## 2 Materials and methods

### 2.1 Pretreatment of RM

**FIGURE 1:**
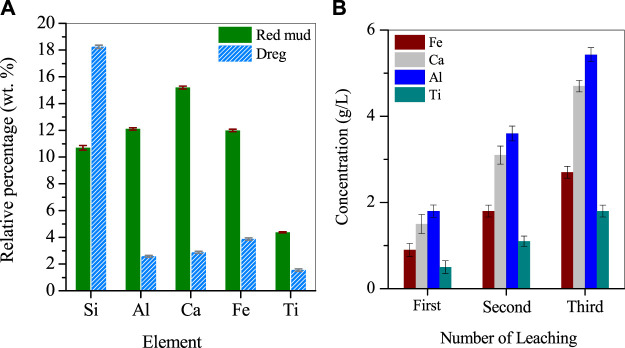
**(A)** Major composition of RM and leached dregs, **(B)** leachate composition.

The RM used in this study was sourced from Hebei Metal Manufacturing. The RM was dried in a vacuum drying oven (Shanghai Jinghong·SZF6090) overnight and stored in a cooling dry box at room temperature. The dried RM was ground to a particle size of about 200 mesh using an agate mortar and stored in a sealed glass bottle in a dry cabinet at room temperature away from light. X-ray fluorescence spectroscopy (XRF) was used to analyze the composition of 1 g of the dried red clay powder, which revealed that it contained mainly 10.69% Si, 12.1% Al, 15.2% Ca, 10.99% Fe, and 4.37% Ti (Figure 1A).

For the metal leaching experiment, 30 g of dried RM and 20 mL of nitric acid were added to a 100 mL glass beaker and stirred on an electric heating magnetic stirrer at 80°C and 90 rpm for 1 h (the leaching experiment was carried out in a fume hood). After stirring, the acid leachate was allowed to cool to room temperature and centrifuged at 8,000 rpm for 5 min to separate the solids and the liquid. The bottom solids were collected after washing twice with ultrapure water and drying overnight at 105°C. The dried solids were ground and analyzed by XRF to determine their composition, which showed that Si was 18.25%, Al was 2.58%, Ca was 2.88%, Fe was 3.87%, and Ti was 1.55%. The supernatant was collected, and another 20 g of RM was added to the supernatant for a second metal leaching using the same method. This process was repeated three times to ensure a high concentration of metals in the leachate (Figure 1B).

The metal acidic leaching solution collected from the previous steps was diluted 5-fold with deionized water and named “Supernatant I". The purpose of dilution was to decrease the concentration of nitrate ions in the solution, which can interfere with subsequent metal separation experiments. Supernatant I was subjected to analysis using inductively coupled plasma emission spectroscopy (ICP), and the concentrations of Fe, Al, Ca, and Ti in the solution were measured to be 2.7 g/L, 4.7 g/L, 5.43 g/L, and 1.8 g/L, respectively, with a pH of −0.25. The solution appeared as a light turbid brown color. Supernatant I was kept refrigerated in a polyethylene bottle for future use.

### 2.2 Precious metals Ti recovery

The recovery process of Ti from Supernatant I was carried out using the following optimized procedure. Firstly, 30 mL of Supernatant I was taken in a 50 mL glass beaker and the pH was measured using a pH meter. The pH of the solution was then adjusted to 0.3 by adding 4 M NaOH solution. Subsequently, varying amounts of sucrose were accurately weighed and added to the beaker containing Supernatant I. The mixture was stirred at 90 rpm for 10 min using a magnetic stirrer before being transferred to a 50 mL reaction vessel. The reaction vessel was then placed into the oven and heated to 60°C for a reaction period of 10 h. After completion of the reaction, the reaction vessel was allowed to cool to around 25°C, and the inner container was removed. The supernatant (Supernatant II) and the bottom precipitate were collected from the inner container.

The white precipitate formed at the bottom of the reaction was washed three times with ultrapure water, dried in a vacuum oven at 105°C for 6 h, and then ground before being stored for characterization as per the previous sample processing method. Parallel comparative experiments were conducted to optimize the reaction time and hydrothermal temperature to achieve the most favorable experimental conditions.

### 2.3 Stepwise recycling of Fe/Al/Ca

After the recovery of Ti, a large amount of remaining metal ions were present in the solution. The residual metal is gradually recovered according to the following method. First, 20 mL of Supernatant II was added to the inner vessel of a 50 mL polytetrafluoroethylene reaction kettle. The inner vessel was then placed in the outer shell of the reaction kettle and heated at 110°C for 3.5 h in a program-controlled temperature box. After the reaction, the reaction kettle was taken out and cooled to room temperature (25°C) in a ventilated area. The brown precipitate generated at the bottom was separated from the supernatant and collected. The collected brown precipitate was dried at 105°C for 6 h in the program-controlled temperature box. The supernatant collected in a centrifuge tube was named as Supernatant III. We also conducted parallel experiments to test the effect of reaction temperature on the efficiency of iron removal.

After removing iron, we continued to remove aluminum from the solution of the Supernatant III by using a hydrothermal method. Specifically, 20 mL of the Supernatant III was added into a 50 mL reactor vessel and heated at 260°C for 20 h to remove aluminum. After the reaction, the solution was subjected to solid-liquid separation using the same procedures as before, and the resulting liquid was named Supernatant IV. The precipitates were dried and characterized according to the previous method.

Following the three-step hydrothermal treatment, titanium, iron, and aluminum were removed from Supernatant I, leaving only calcium ions in the solution. To remove calcium from the Supernatant III, we adjusted the pH using concentrated sulfuric acid and continuously stirred the solution at 100 rpm for 20 min until the pH value of Supernatant IV reached 0.1. We collected the resulting white precipitate, which was then dried overnight at 70°C in a programmable oven. Thus, the metal solution obtained from the acid leaching of RM was gradually processed and corresponding by-products were obtained.

## 3 Results and discussion

### 3.1 Effective leaching of Ti from RM

**FIGURE 2:**
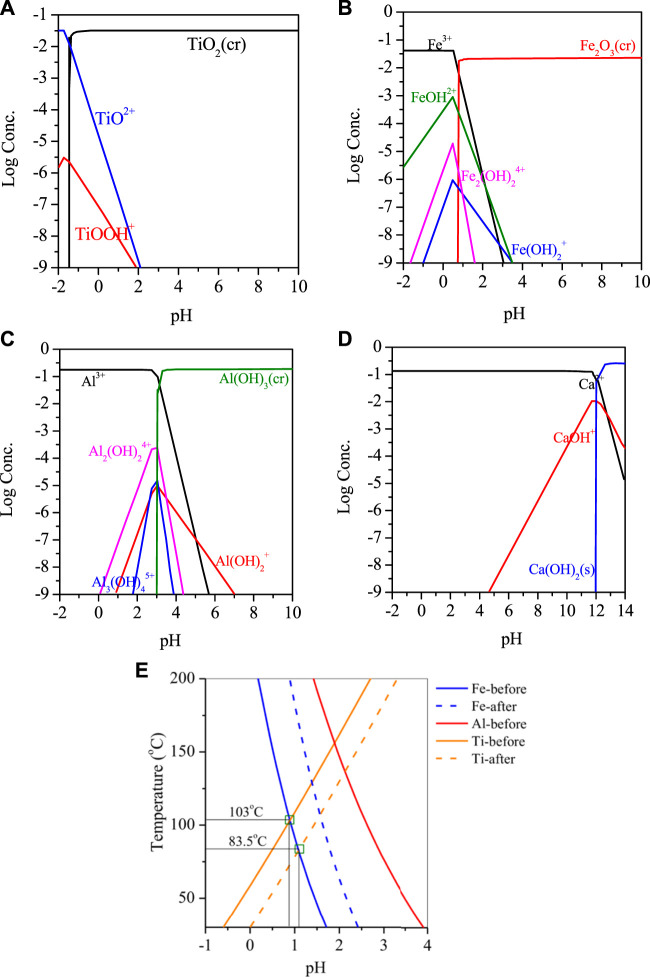
Hydrolysis plot of **(A)** Ti, **(B)** Fe, **(C)** Al, and **(D)** Ca versus pH, **(E)** pH-T relationship of Ti/Fe/Al hydrolysis in RM.

RM is a mixture of hematite, gypsum, katoite, kaolinite and contains about 4.5% Ti. When the RM was leached with nitric acid solution multiple times, the metal concentration in the solution increased with the number of leaching cycles. After the third cycle, the concentrations of Fe, Al, Ca, and Ti in the solution reached 2.76 g/L, 4.72 g/L, 5.43 g/L, and 1.88 g/L, respectively. Considering the metal efficiency and the concentration of nitrate ions in the solution, leaching for 3 cycles was determined as the optimal condition. For the insoluble residue remaining in the leached RM, analysis showed that it mainly contained a large amount of oxidized Si and small amounts of Fe, Al, Ti. The residue could be further leached for metal recovery.The reaction was simulated by HSC software and Medusa. HSC Chemistry software (9.5, Metso Outotec, Finland) and Medusa software (1.0, Royal Swedish Institute of Technology, Switzerland). Based on Figures 2A–E, it is evident that Ti, Fe, and Al present in the acidic leachate were reactive cations, which underwent hydrolysis when the pH was greater than −1, 0.7, 2.6, and 11.8. This resulted in the formation of their respective hydrated cations, such as TiOOH^+^, FeOH^2+^, Al_2_(OH)_2_^4+^ and CaOH^+^. However, adjusting the pH can cause co-precipitation of these metals, which can result in a lower purity of the final product. During the temperature increase process, the hydrolysis of Ti in the solution showed a different trend compared to Fe/Al, with two distinct points. The first difference lies in the hydrolysis product of Ti, which is mainly TiO(OH)_2_ instead of the unstable Ti(OH)_4_. The presence of TiO(OH)_2_ can promote the formation of aggregates rapidly, which is also the main purpose of adding Ti to Fe/Al coagulants on the market. The second difference is the effect of temperature. As the temperature increased, the pH value at which hydrolysis occurs gradually increased, indicating that increasing the reaction temperature inhibited the hydrolysis of Ti. Theoretical calculation results showed that when the pH value is constant, the Gibbs energy constant for the formation of TiO(OH)_2_ at room temperature is −23.2 kJ/mol, and when the temperature is raised to 100°C, the constant increases to 2.94 kJ/mol, indicating that the reaction cannot continue automatically under this condition.

### 3.2 Verification experiment of Ti hydrothermal separation

**FIGURE 3:**
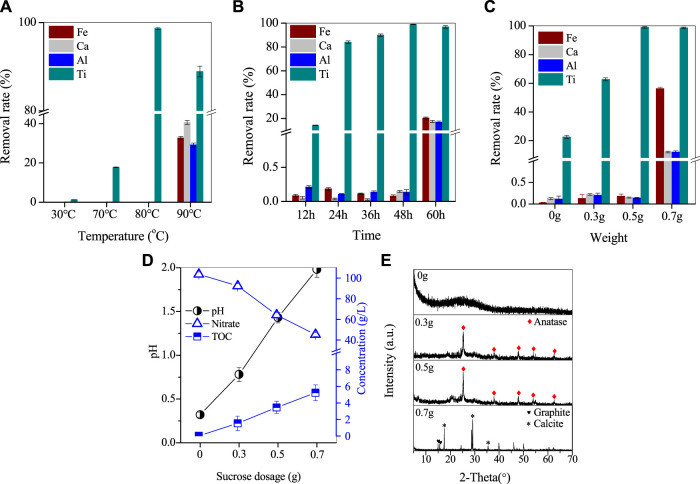
Removal of Ti by varying **(A)** the temperature, **(B)** the reaction time, and **(C)** the organics dosage, **(D)** the corresponding variation of pH, nitrate and TOC by adding organics, **(E)** XRD patterns.

In this study, experiments were conducted to investigate the preferential removal of Ti, with a focus on the effects of temperature, time, and different organic reagents on the process. To promote Ti separation, sucrose was added to the reaction solution, and the results were presented in Figure 3A. After 48 h of reaction at room temperature, Ti remained stable in the leaching solution without undergoing hydrolysis. However, increasing the reaction temperature up to 70°C resulted in the hydrolysis of Ti, with nearly 17.8% Ti removed from the solution while the loss of other elements was less than 0.15%. By further increasing the temperature to 80°C, the removal rate of Ti significantly increased to 98.6%, while the loss rate of other elements remained negligible. At 90°C, the removal rate of Ti decreased to 88.9%, and the removal rates of Fe/Al/Ca reached 32.7%, 40.7%, and 29.2%, respectively. The co-precipitation of metal ions at this temperature may be attributed to the high temperature promoting the redox reaction of sucrose and nitrate, thus consuming excess H^+^ and causing the solution pH to increase. This increase in pH may then promote the hydrolysis of Fe/Al hydroxides (Eq. 4).

To examine the effect of reaction time on Ti hydrolysis, the Ti removal rate was studied in Figure 3B. At 80°C and a sucrose dose of 0.5 g, the Ti removal rate was only 14.2% in 12 h. However, as the reaction time was extended from 12 h to 48 h, the Ti removal rate gradually increased from 14.2% to 98.6%, while the loss of Fe/Al/Ca was less than 0.2%. By continuing to extend the reaction time to 60 h, the removal of Ti slightly decreased to 96.8%, but the removal of Fe/Al/Ca was nearly 20%. The pH increase caused by poor pH control of the reaction solution led to the coprecipitation of metal ions, which is similar to the aforementioned increase in temperature to 90°C.

The effect of sucrose dose on Ti separation was investigated, and the results are presented in Figure 3C. Without the addition of sucrose, the Ti removal rate after 48 h of reaction at 80°C was only 22.6%. However, with the addition of 0.3 g of sucrose, the Ti removal rate increased significantly to 62.8%, and further increasing the sucrose dose to 0.5 g resulted in a Ti removal rate of 98.6% with a loss rate of Fe/Al/Ca less than 0.15%. Nevertheless, increasing the sucrose dose to 0.7 g did not significantly affect the Ti removal rate, but the removal rates of Fe/Al/Ca increased significantly to 56.2%, 12.1%, and 12%, respectively.

The process of Ti removal by sucrose dose was analyzed by analyzing the nitrate concentration, TOC and solution pH in the supernatant after the reaction, and the results are shown in Figure 3D. The initial pH of the RM leach solution was −0.15, and the reaction was started at a constant temperature after the pH was adjusted to 0.3 by using NaOH. After 48 h, the pH of the solution increased slightly to 0.32, while the nitrate concentration in the solution decreased from 109.4 to 103.6 g/L. The increase of pH in the solution was found to be mainly related to the thermal decomposition of the high nitrate concentration. In this process, a part of the solution H^+^ and H^+^ produced by the hydrolysis of Ti, was consumed in this reaction, leading to a slight increase in the pH of the solution, which can be elucidated. The addition of 0.3 g of sucrose further increased the pH of the solution to 0.8, while the nitrate concentration decreased from 103.6 to 92.2 g/L. Sucrose is an organic substance whose addition to the solution significantly increased the TOC value. By comparing the changes in TOC before and after the reaction, it was found that the TOC consumed during the addition of 0.3 g of sucrose also reached 1.5 g/L. Although sucrose undergoes a warming hydrolysis in acidic solutions to produce sucrose and fructose, this does not significantly reduce the TOC value of the solution. Therefore, the depletion of TOC is clearly due to the oxidation of organic matter, and it can be judged that the reaction between sucrose and nitrate in this experiment is the main reason for the continued increase in pH of the solution.

After increasing the sucrose dose to 0.5 g, the solution’s pH continued to increase to 1.5, and the nitrate concentration and consumed TOC reached 64.3 and 3.5 g/L, respectively. As a result, almost 98.6% of Ti underwent hydrolysis and crystallization, resulting in its removal from the solution. However, the loss of Fe/Al/Ca was minimal at less than 0.15%. Once the sucrose dose reached 0.7 g, the solution’s pH increased to 2, and the nitrate concentration further decreased to 45.3 g/L, while the consumed TOC increased to 5.2 g/L. At this point, the removal of Ti/Fe/Al/Ca occurred simultaneously.

**FIGURE 4:**
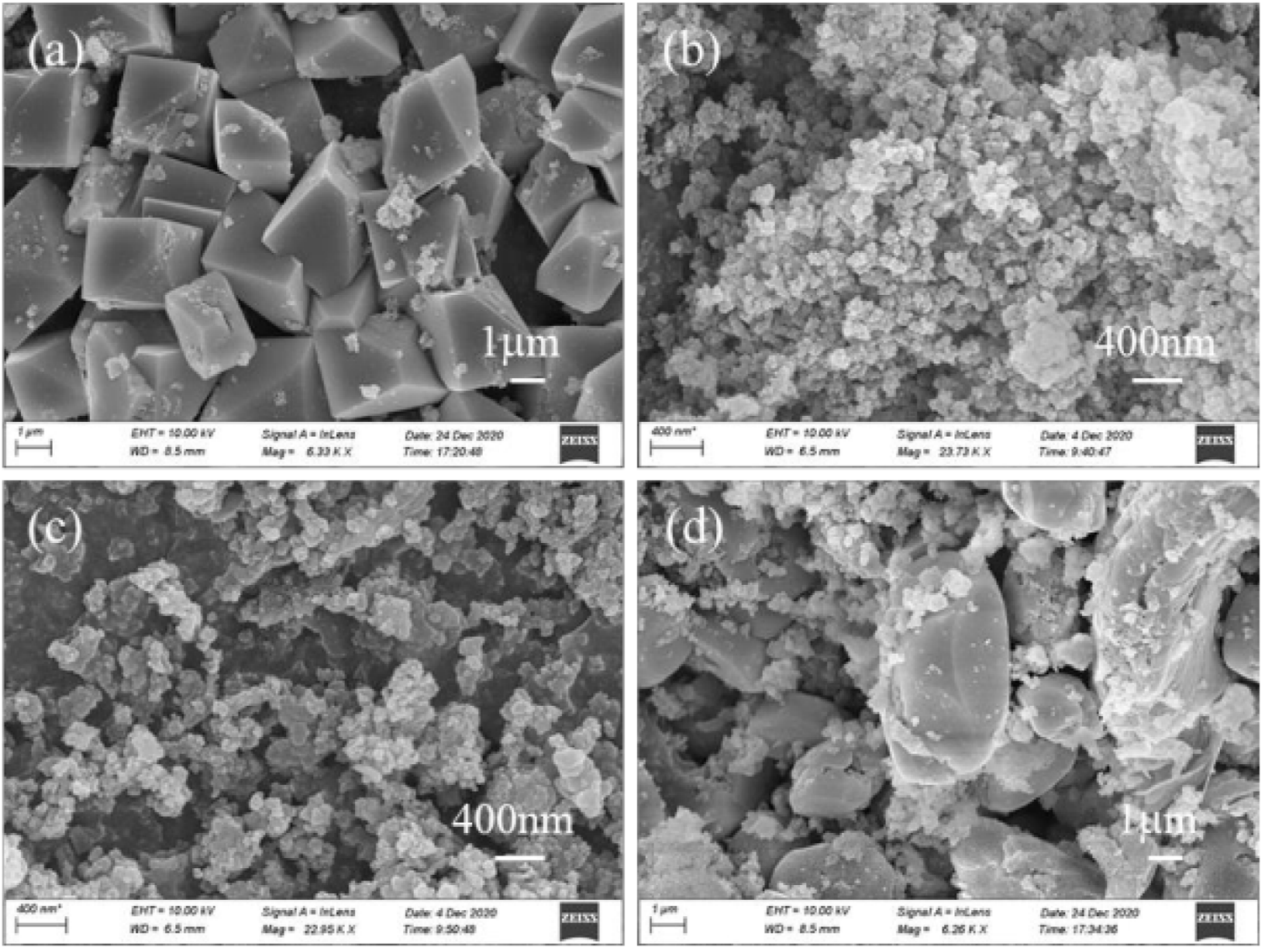
Removal of Ti by varying SEM images of the corresponding products.

Additionally, the Ti-containing product obtained from the above process was analyzed further. The XRD spectrum of the Ti-containing product without sucrose exhibited a broad diffraction peak (Figure 3E), indicating that the product was a weakly crystalline Ti-containing mineral. The product showed massive agglomeration with a smooth surface and neat edges, indicating a Ti-containing hydrate (Figure 4A). When 0.3 and 0.5 g of sucrose were added, the corresponding products Figures 4B, C showed spherical particles of 400 µm in size with a few massive agglomerates in SEM. However, when the dose was increased to 0.7 g, the XRD spectra of the products showed the disappearance of anatase characteristic peaks, while two broad peaks appeared between 20°–40° (Figure 3E). These peaks corresponded to Ti/Fe hydrates, with a large number of smooth surface masses on their SEM patterns (Figure 4D).

### 3.3 Continued separation of Fe, Al, and Ca

**FIGURE 5:**
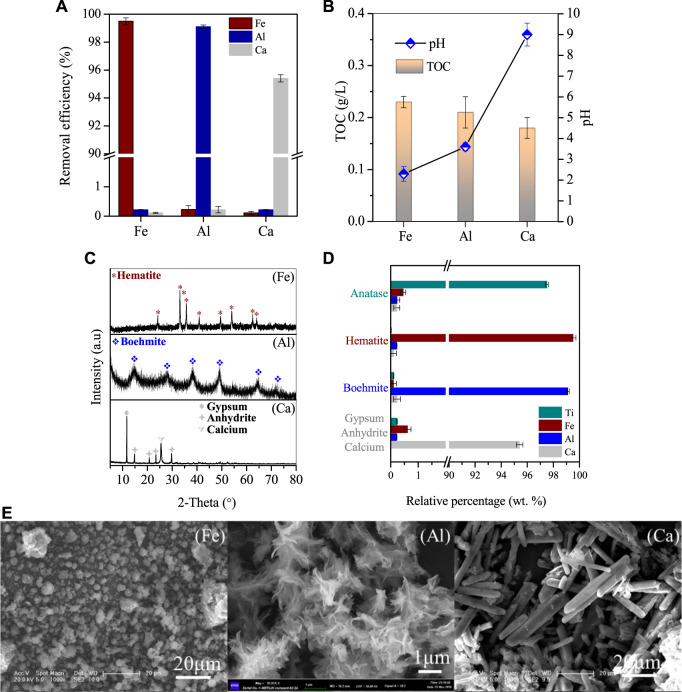
Stepwise removal of Ca, Fe, and Al. **(A)** Removal efficiencies, **(B)** pH value and TOC variations, and **(C)** XRD patterns and **(E)** SEM images of the corresponding generated deposits, **(D)** major compositions of the products generated at the optimized condition, The Ca in the supernatant was further processed with sulfuric acid, and the residue was 10.25 mg/L, indicating that 97.6% of the Ca precipitated into anhydrite nanorods with a diameter of 2–10 μm and a length of 30–50 μm (Figure 5E). We have characterized the products obtained from resource recovery and found that their purity is relatively high (Figure 5D). Specifically, the content of Ti in Anatase reaches 97.5% with trace amounts of Fe, Al, and Ca ions. Similarly, the purity of the other by-products, namely, hematite, boehmite, and anhydrite, is also above 95%. These by-products can be used as raw materials for industrial manufacturing.

After Ti was removed, the pH of the supernatant increased to 1.5 and was further collected for the recovery of Fe/Al/Ca. In the supernatant, the residual concentration of Ti was only 16.8 mg/L, while the concentrations of Fe/Al/Ca were 2.66, 4.65, and 5.41 g/L, respectively. When Supernatant II is directly heated to 110°C for 4 h, 99.5% of Fe in the solution is removed, while the loss of Ca and Al is only about 0.3% (Figure 5A). With the removal of Fe, the pH of the solution also increased from 1.5 to 2.3, while the TOC value in the solution also decreased simultaneously. This indicates that sucrose in the solution continued to hydrolyze at high temperatures. The separated iron was detected as 1 μm spherical hematite particles with a few aggregates (Figures 5B, C). After removing the iron, the concentration of Fe in the solution was 5.1 mg/L, while the concentrations of Al/Ca were 4.57 g/L and 5.39 g/L, respectively, showing that Al/Ca remained stable in the solution and did not participate in the hydrolysis and co-precipitation of Fe.

Continuing the reaction of Supernatant III, the reaction was carried out under conditions of 270°C for 20 h 99.1% of the Al in the solution was successfully removed, and the product was characterized as Boehmite by XRD (Figures 5C, D (Al)). Multiple prominent peaks of Boehmite appeared continuously at 2θ = 15.2° in the figure, with good peak shape and no other impurities present. At the same time, through SEM characterization, it can be seen that the morphology of the product in Figure 5E (Al) is a small flake, with layers of flakes gathered in a collective composed of one piece, and the single size of the flake is between 1–2 μm. After the reaction, the pH of the solution increased to 3.6, and the nitrate ion content was around 450 mg/L. The residual Al in the solution was 8.6 mg/L, and the concentration of Ca was 5.39 g/L.

### 3.4 Separation mechanism

Upon dissolution of RM powder with nitric acid, the metal ions including Ti, Fe, Al and Ca present in the RM are effectively solubilized. By employing a combination of water bath heating and stirring, a solution with high concentration of Ti, Fe, Al, and Ca is obtained, along with a residual Si-rich residue that remains insoluble (Kazak, 2021). The presence of SiO_2_ in the RM effectively isolates Si from the reaction with concentrated nitric acid, thereby precluding its dissolution. Consequently, Si is retained in the RM and separated in the form of an insoluble residue (Eq. 1).

After initial data analysis, it is evident that temperature increase in the solution hindered Ti hydrolysis, but sucrose was added in this experiment to control pH value. The reaction between sucrose and nitrate consumed H^+^ and facilitated Ti hydrolysis (Eq. 4). Ti hydrolysis follows a time course, reaching an optimal value at 48 h. Higher hydrothermal reaction temperature was found to promote Ti hydrolysis but also led to Ti/Fe co-precipitation (Li et al., 2023). Ti hydrolysis and crystallization releases H^+^ and the Gibbs constant of this reaction is −23.2 kJ/mol at room temperature, indicating automatic Ti hydrolysis at a suitable pH (Eq. 2). H^+^ accumulation lowers solution pH, which, in the absence of nitrate, causes a large amount of residual Ti in solution and reduces Ti removal efficiency. Under heating conditions, nitrate decomposes into NO_2_, O_2_, and H_2_O, consuming H^+^ and promoting Ti hydrolysis. The Ti-containing microcrystals bond together by H/H_2_O bonds, forming a complex meshwork.

Fe hydrolysis produces Fe-containing hydroxides and generates H_2_O that consumes H+, raising solution pH. Reaction temperature was controlled at 110°C in Fe reaction to counter initial pH increase. Nitrate-sucrose redox reaction rate increases rapidly at high temperature, and pH of supernatant III increased from 1.48 to 3.2. In the hydrothermal system, Fe^3+^ hydrolyzes with hematite to form iron oxyhydroxide, releasing a large amount of H^+^ into the liquid phase (Eq. 3). H^+^ accumulation leads to equilibrium, with high Fe^3+^ content in solution. Sucrose consumption facilitated nitrate-sucrose redox reaction, allowing Fe and Al hydrolysis to continue, reducing residual Fe and Al content to 5.1 mg/L and 8.6 mg/L (Eqs. 5, 6). Calcium precipitation followed ordered separation (Eq. 7). Under acidic conditions, cations such as Ti^2+^, Al^3+^, and Ca^2+^ can coordinate with surface hydroxyl groups on Fe oxyhydroxides. H^+^ competes with cations on the surface of Fe oxides for hydroxyl sites, allowing Ti^2+^, Al^3+^, and Ca^2+^ to remain in the liquid phase under acidic conditions.

$$Si+O_{2}\;\to \;{SiO}_{2}$$ (1)

$${Ti}^{4+}+3H_{2}O↔TiO{\left(OH\right)}_{2}+4H^{+}$$ (2)

$$Fe^{3+}+3H_{2}O\to Fe{\left(OH\right)}_{3}+3H^{+}$$ (3)

$$88H^{+}+5C_{22}H_{22}O_{11}+88NO_{3}^{-}\to 110CO_{2}+99H_{2}O+44N_{2}$$ (4)

$$2Fe{\left(OH\right)}_{3}\to 2FeOOH+2H_{2}O\to {Fe}_{2}O_{3}+{3H}_{2}O$$ (5)

$${2Al}^{3+}+6H_{2}O\to 2Al{\left(OH\right)}_{3}+6H^{+}\to 2AlOOH+2H_{2}O+6H^{+}$$ (6)

$$Ca^{2+}+SO_{4}^{2-}\to CaSO_{4}$$ (7)

### 3.5 Mass balance analysis

**FIGURE 6:**
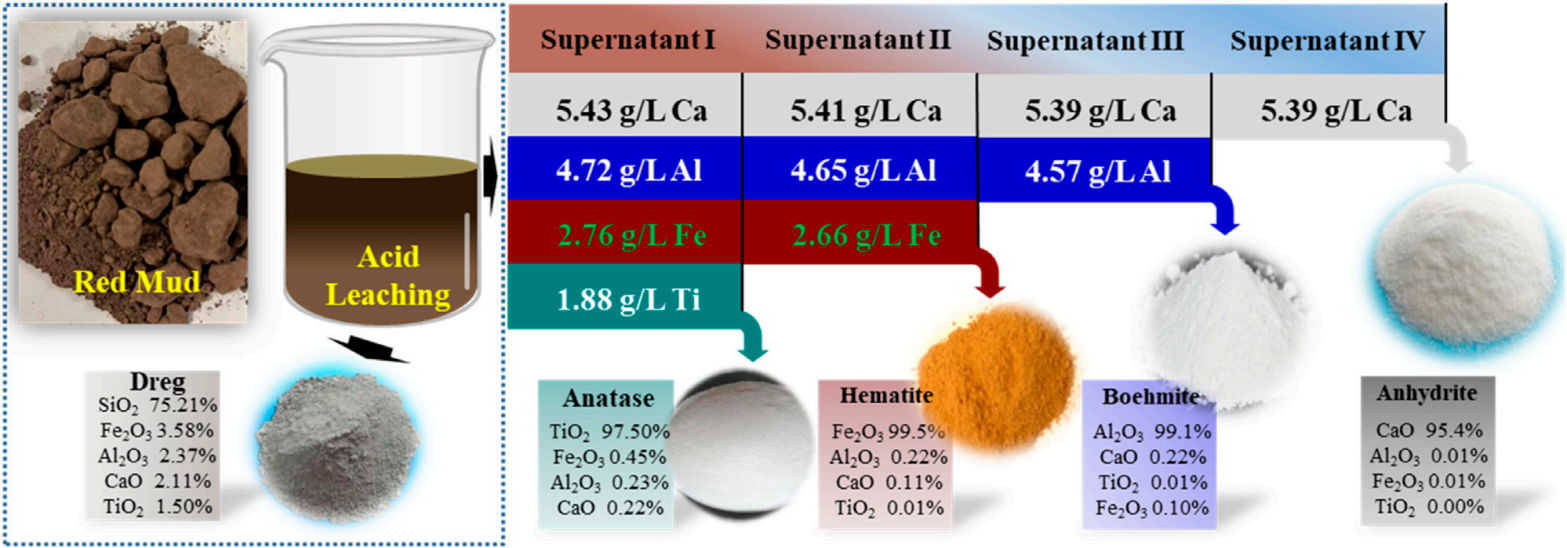
Mass balance of Ti, Fe, Al, and Ca separation from waste RM.

The mass balance of the entire separation process for Ti, Fe, Al, and Ca recovered from RM is illustrated in Figure 6. By introducing organic matter, 98.6% of titanium was selectively separated from the acidic leach solution in the form of anatase, which contained 97.5% TiO_2_. Moreover, the recovery of Fe, Al, and Ca in solution reached 99.5%, 99.1%, and 97.6%, respectively, after the Ti separation and recovery process. The corresponding products obtained were 99.5% pure hematite, 99.1% boehmite, and a mixture containing 95.4% gypsum, anhydrite, and calcium. The other insoluble matter of the untreated hematite is mainly silicon-dominated, accounting for about 5% of the hematite, approximately 1.5 g, which can be further utilized as a resource. As a result, the RM was successfully recycled through four steps, reducing the pressure of mining and promoting the reuse of RM. Therefore, the future solid waste disposal market holds promising development prospects.

The consumption of resources in the process was calculated and it was found that one ton of RM required approximately 0.25 tons of nitric acid, 0.12 tons of sucrose, 0.12 tons of tap water, and 48 kW·h of electricity for the reactor (Table 1). In contrast, the process yields 0.11 tons of Anatase, 0.15 tons of Hematite, 0.25 tons of Boehmite, and 0.34 tons of Anhydrite. The economic value of the recovered products far exceeds the cost required for the disposal of RM, making the recovery process an effective method of waste resource utilization.

**TABLE 1:** Economic analysis of Ti, Fe, Al, and Ca recovery.

	Reagent and energy	Price (USD/t)	Usage/product (t)	Subtotal price (USD/t)
Reagent and energy consumption	Nitric acid	319.1	0.25	79.78
	Sucrose	158.66	0.12	19.03
	Electric energy	0.15	48	7.2
	Tap water	0.44	0.12	0.0528
Major product	Anatase	1850	0.07	129.5
	Hematite	150	0.15	22.5
	Boehmite	200	0.20	40.0
	Anhydrite	38	0.24	9.12
Total cost				101.06

In the table, the reagent price was acquired from b2b.baidu.com, whilst the water price was recorded in the website of https://www.ahsz.gov.cn/content/column/167469421.

## 4 Conclusion

The current landfill disposal method for RM, a toxic and hazardous waste, fails to recycle the valuable metal resources it contains, resulting in significant waste. RM contains a significant amount of strategic metal, titanium. In order to promote the resource utilization of RM, we developed a leaching coupled with hydrothermal method to recover the metal ions present in it. Through a tertiary leaching process, an acidic leachate was obtained with concentrations of metal ions Fe, Al, Ti, and Ca of 2.7 g/L, 4.7 g/L, 5.43 g/L, and 1.8 g/L, respectively. Subsequently, 99.3% of the Ti was recovered as anatase nanoparticles by introducing sucrose at 80°C. Following this, Fe was recovered from the leachate as hematite after reacting for 4 h at a temperature of 110°C. Further hydrothermal treatment was used to recover Al in the form of boehmite. Finally, the pH was adjusted to recover 97.6% of Ca as a mixture of gypsum, anhydrite, and calcium. The final concentration of metal ions in the solution was less than 10 mg/L. This method offers several advantages for the resource recovery of RM, including: 1) reducing the pressure of landfill disposal by over 90%, 2) obtaining high purity products (above 95%) with certain market economic value, and 3) generating revenue of approximately 100 USD per ton of RM according to economic calculations. In conclusion, our leaching coupled with hydrothermal method is an effective way to recover valuable metal ions from RM, providing significant economic and environmental benefits.

## Data Availability

The original contributions presented in the study are included in the article/Supplementary Material, further inquiries can be directed to the corresponding author.
